# Bidirectional and cross-lag relationship between social media use and psychological wellbeing: evidence from an Indian adolescent cohort study

**DOI:** 10.1186/s12889-023-17276-1

**Published:** 2024-01-26

**Authors:** Chanda Maurya, Preeti Dhillon, Himani Sharma, Pradeep Kumar

**Affiliations:** 1 Department of survey research and data analytics, International Institute for Population Sciences, Govandi Station Road, Mumbai, 400088 India; 2 International Institute for Population Sciences, Mumbai, Govandi Station Road, Mumbai, 400088 India

**Keywords:** Social Media, Depressive symptoms, Adolescents, Longitudinal study, Cross-lagged model

## Abstract

**Introduction:**

In an online era like today, the relationship between social media and depression among adolescents and young adults is erratic and still continues to be a debatable subject. The study aims to examine the association and bi-directional relationship between social media usage and depressive symptoms among the adolescent boys and girls in India.

**Methods:**

The study uses data from two waves of Understanding the Lives of Adolescents and Young Adults (UDAYA) project survey conducted in two Indian states of Uttar Pradesh and Bihar. Depression was measured by a Patient Health Questionnaire. Logistic regression has been used for analyzing the data comprising the same time period, whereas the bidirectional relationship between two time periods has been evaluated by Cross-Lagged Path Model.

**Result:**

Findings suggest that the percentage of moderate depressive symptoms increased from 1.7% to 3.0% from Wave 1 to Wave 2. Depression among adolescent girls increased slightly from wave 1 to wave 2 whereas a slight decrement was noticed in the moderate form of depressive symptom among adolescent girls using social media for the two waves. Socioeconomic factors like education, age, gender played an important role in affecting depression among adolescents in both the Wave of the survey. The path relationship reveals that social media users in Wave 1 [β=0.22, *p*<0.001] were positively associated with social media users in Wave 2. Similar patterns were observed for depressive symptoms at both the waves of the survey. However, cross lagged relations between social media use and depression could not be established between the survey periods.

**Conclusion:**

A significant degree of association was found between social media use and depression among adolescent boys and girls in the study. The present study concludes that factors like age, gender and education showed significant relationships with social media use and depression.

**Supplementary Information:**

The online version contains supplementary material available at 10.1186/s12889-023-17276-1.

## Background

Internet is becoming a widely accepted channel for networking as well as for the exchange of information worldwide. Globally, there are rapid growth and development in its numbers and size[1, 2], particularly among adolescents [3]. Worldwide, approximately two million people access the internet. From 2000 to 2012, the growth rate of internet usage increases up to 566.4%. Asia has the highest number of Internet users globally, which is approximately 922.3 million, representing 44% share of the world's Internet user population, as cited by Internet World Stats [4]. The use of information technologies has shown rapid growth during the last decade in almost every country globally. Increasing computer ownership and internet access have changed millions of people's lives [5]. They go online to send/receive emails, chat, research for school or work, download music or images and do many other activities [5]. Almost 90 percent of young adults with internet access used social media [6]. Any web site that allows social interaction is considered a social media site [7]. Such sites include Facebook, Instagram, myspace, Youtube, Snapchat, and Twitter, to name a few; however, new sites are continually emerging. People can use for a variety of activities designed to satisfy basic social needs, including chatting with friends, sharing photos and videos, blogging, dating, meeting new people, entertainment, health promotion [8], and gambling [7, 9]. In 2021, approximately 4.26 billion people were using social media, and as projected, this number would become up to 7 billion in 2027 [10]. With the ease of internet access, In India, there was 326.1 million social media user in 2018. However, these numbers are comparably lower than those that took place in 2016 and 2017.

Users of social networking sites were 518 million in 2020 in India, expected to be 1.5 billion in 2040, on the platforms like Facebook, the largest social media platform, followed by YouTube [11] and WhatsApp [12]. Adolescents and young adults are the most active social media users, which have a predominantly high risk for developing mental health issues [13–15]. Many studies implicate social media use increases mental distress. According to a study conducted in North America, mental health has paralleled a steep rise in smartphones and social media use by children and adolescents [16]. Social networking sites are now a normative part of adolescent development [17]. However, with time, the scenario is changing in the developing world too. Numerous plausible potential intervening pathways related to young people's mental health to the amount of time they spend on social networking sites and how they engage themselves and interact online [14]. Social media's excessive use can affect adolescents' self-view and interpersonal relationships through social comparison and hostile interactions, including cyberbullying, online harassment, poor sleep, low self-esteem, poor body image, and physical activity. In the Indian context, the rampant use of social media among adolescents has led to detrimental consequences. Constant social comparison exacerbates pressure in a society already laden with expectations. Cyberbullying and online harassment have become pervasive issues, causing emotional distress. Moreover, excessive screen time disrupts sleep patterns and promotes sedentary lifestyles, contributing to physical and mental health challenges, including low self-esteem and poor body image. This complex interplay of factors underscores the urgent need for digital literacy and mental health awareness in India's youth [14, 18]. These are the potential mediators which affect psychological wellbeing. Heavy internet or social media use is associated with media multitasking. Increased media multitasking was associated with higher depression and social anxiety symptoms, even after controlling for general media use [19]. It may be problematic for some youth but also beneficial for others. There may be a convergence between the subjective belief of tech intensity and multitasking behavior [20]. Multitasking media with symptoms was reflected by changes in the functioning due to depressed mood or loss of interest and impaired work or social functioning [21]. Media multitasking during entertainment activities was correlated with increased social success, normalcy, and self-control [22]. The dependency of youths on social media has reached such a level that, without social media, every young person cannot think about the direction of their growth [23]. Social media use for chatting, emailing, participating in communities or clubs, and using bulletin boards negatively affects mental health and suicidal ideation [24].

Age and sex are significant predictors of social media use. There are big differences in social media use among males and females in the adolescent age group. High levels of social media interaction in early adolescence have implications for wellbeing or depression in later adolescence, particularly for females [13, 14]. According to Heffer et al., social-media use did not predict depressive symptoms over time for males or females[25].

Person’s behavior play an important role in shaping the social-psychological and emotional well-being. Studies found that those who uses substance were at higher risk of having mental health problem than the non-users [26]. Adolescent’s psychological well-being depends on the socio-economic status of the family (i.e., family income, parental educational level, caste, religion) [27]. One safeguard for the family is the mother's education, which is linked to other favorable family traits. Numerous research revealed that kids with higher levels of schooling have much less mental health issues [27, 28]. Adolescents from lower social group had higher chance to get mental health problems[29, 30].

However, more significant depressive symptoms predicted more frequent social-media use only among adolescent girls. However, Puukko et al., shows that the depressive symptoms predicted small increases in active social media use during early and late adolescence[31]. In contrast, no evidence of the reverse relationship was found. Some studies show the bi-directional relationship between social media use and psychological wellbeing [32, 33].

This bidirectional relationship between social media use and depressive symptoms can be explained through two primary theoretical approaches [3, 34, 35]. The first is the displacement approach, which explains that the time spent on social media happens at the expense of other activities related to a person's overall wellbeing, such as physical activities, playing games, or spending time with friends or family. According to this theory, time spends on social media might be related to depressive symptoms among adolescents. The second is the social compensation approach which advocates that social media can act as a healer to those adolescents with pre-existing mental health problems, depression, or perceived psychological problems. Social media, in this case, is used by an adolescent as a form of escapism from the existing distress and depressive symptoms by establishing a connection with others on a virtual platform [31].

Research on the impact of social media use on mental health often comes from Western contexts, and there is a limited understanding of how these trends manifest in developing countries. Given the rapid growth of internet and smartphone penetration in India, this study is particularly relevant to understanding the dynamics in emerging economies. The study recognizes a critical gap in existing research, which primarily consists of cross-sectional studies. Cross-sectional research provides a snapshot of a particular point in time but lacks the ability to capture changes and trends over time. This study seeks to overcome this limitation by conducting longitudinal research, tracking changes in social media use and its impact on psychological wellbeing over a 3-year period. The study's geographical focus on two populous and economically diverse states in India, Uttar Pradesh and Bihar, adds uniqueness. The use of a state representative sample is crucial. Uttar Pradesh and Bihar are two of India's largest and most diverse states, and they provide a comprehensive picture of the country's social and economic diversity. This approach enhances the generalizability of the findings to a wider population.

The study's focus on the transition from adolescence to early adulthood is a key strength. This is a critical and understudied period of life characterized by significant social, emotional, and psychological changes. Understanding how social media use affects mental health during this transition is vital for developing targeted interventions. By narrowing the study to unmarried boys and girls, the research recognizes a potentially vulnerable group. Adolescents and young adults who are not married may be more likely to spend significant time on social media, making them an important subgroup to study. This focus allows for a more nuanced analysis of the relationship between social media use and depression within this specific demographic. The study's combination of both between-subject and within-subject analyses is a methodological strength. This approach allows for a comprehensive examination of the relationship between social media use and depression. The first part assesses the immediate impact, while the second part investigates the bidirectional relationship over different developmental stages, providing a holistic understanding of this complex issue [18–22].

## Data and methods

The data used for analysis was taken from the two-wave longitudinal study "Understanding the Lives of Adolescents and Youth Adults" conducted in Uttar Pradesh and Bihar by the Population Council under the guidance of the Ministry of Health and Family Welfare, Government of India. The survey collected detailed information on education, economic activity, household work, migration, mass media and social media exposure, growing up, aspirations, agency, gender role attitudes, awareness of sexual and reproductive health matters, romantic and sexual relationships, and health-seeking behavior etc. The UDAYA adopted a multi-stage systematic sampling design to provide the estimates for states as a whole and urban and rural areas of the states [36]. The study has been designed to establish the levels, patterns and trends in the situation of younger (10-14) and older (15-19) adolescents, conducted in 2015–16 (Wave 1), and the follow up of participants conducted to shed light on the factors that determine successful transitions to adulthood, about three years later when they were aged 13–22 in 2018–19 (Wave 2). The analytical sample consists of both younger (10−14 years) and older (15−19 years) adolescents who were interviewed at the baseline in 2015-16 and followed-up in 2018-19 at the age (13-17 years) and (18-22 years) respectively [36]. Ethical approval was obtained from the Institutional Review Board of the Population Council. Written consent was obtained from the respondents in both waves. In wave 1 (2015–2016), 20,594 adolescents were interviewed using the structured questionnaire with a response rate of 92%. Moreover, in wave 2 (2018–2019), the study interviewed the participants who were successfully interviewed in 2015–2016 and who consented to be re-interviewed. Of the 20,594 eligible respondents for the re-interview, the survey re-interviewed 4567 boys and 12,251 girls (married and unmarried) with the attrition rate 18.3%. The attrition rate is the percentage of people who stop responding to surveys. After excluding the respondents who gave an inconsistent response to age and education in the follow-up survey (3%), the final follow-up sample covered 4428 boys and 11,864 girls with a follow-up rate of 74% for boys and 81% for girls. The effective sample size for the present study was 4428 adolescent boys and 11,864 adolescent girls aged 13–23 years in wave-2. The cases whose follow-up was lost were excluded from the sample to strongly balance the dataset and set it for longitudinal analysis using the *xtset* command in STATA 14.

**Fig. 1:**
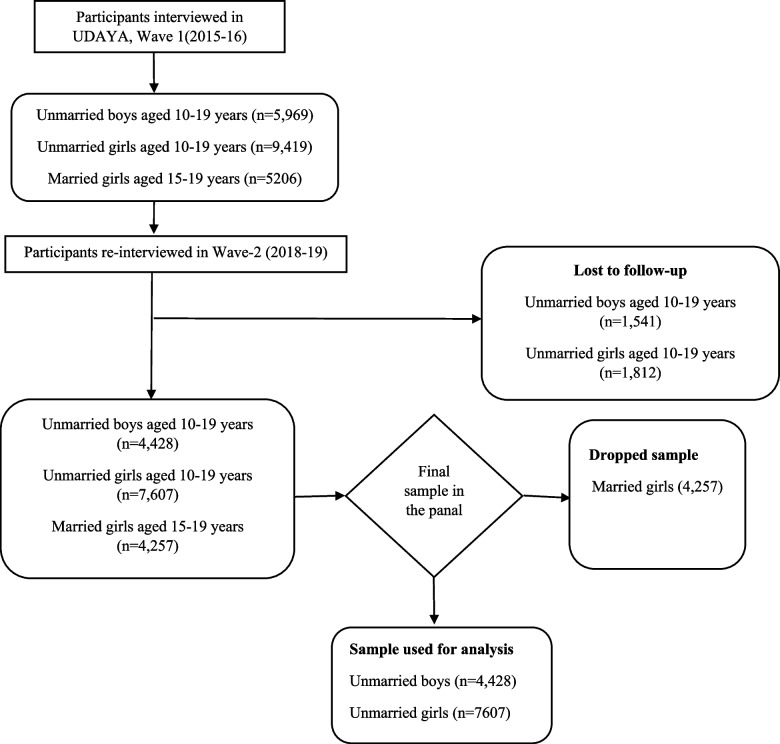
Sample selection criteria

 We restricted our analysis to unmarried and present results for boys and girls separately based on sample distribution of the variable of interest. The adequate sample size for this study was 12035 (4428-boys and 7607-girls) adolescents aged 10-19 at Wave 1. Detailed information about the sample selection criteria is shown in Fig. 1.

### Outcome variable

Depressive symptoms were assessed by asking nine questions from the respondents; the respondent was asked about the symptoms for the past two weeks only. The questions included, (a) had trouble falling asleep or sleeping too much, (b) feeling tired or having little energy, (c) poor appetite or eating too much, (d) trouble concentrating on things, (e) had little interest or pleasure in doing things, (f) feeling down, depressed, or hopeless, (g) feeling bad about yourself, (h) been moving or speaking slowly, (i) had thoughts that respondent would be better off dead. All the above questions were asked on a scale of four, i.e., 0 “not at all,” 1 “less than once in a week,” 2 “one week or more” and 3 “nearly every day.” The scale of 27 points will then generated using the egen command in STATA 14 [37]. The same method was assessed for both the Wave depression symptoms with Cronbach alpha value of 0.85 and 0.81 at Wave 1 and Wave 2, respectively. The variable were recorded in to 4 categories i.e. (a) No depression (0-4), (b) Mild (5-9), (C) Moderate (10-14) and (d) Severe (15-27). Severe includes moderately severe and severe. The categories were redefined for analytical purpose.

### Predictor variables

Different socio-demographic variables including age (10-14 and 15-19 at baseline and 13-16 and 17-22 at follow-up), Sex (adolescent boys and adolescent girls), mother’s education (Not educated and educated), wealth index (poorest, poorer, middle, richer richest), measured at Wave 1.

Index scores were constructed ranged from 0 to 57. Households were then ranked according to the index score. The first quintile represents the households of the lowest (poorest) wealth status and the fifth quintile represents the households with the highest (wealthiest) status. Wealth quintiles were developed at the state level on the basis of the weighted sample for the whole state.

The survey measured household economic status, using a wealth index composed of household asset data on ownership of selected durable goods, including means of transportation, as well as data on access to a number of amenities.

The wealth index was constructed by allocating the following scores to a household’s reported assets or amenities, Type of house, Agricultural land owned, Irrigated land owned, Access to toilet facility, Cooking fuel used:, Access to drinking water facility, Access to electricity and Ownership of household assets.

Current schooling (never attended, dropout and currently attending), substance use (yes and no) and paid work in the last 12 months (yes and no), measured at Wave 1 and Wave 2 by using the same questions. Social media use was assessed by asking question to the respondents “Have you ever used social media, for example, facebook, whatsapp, twitter or we-chat”. It was coded as 1 “yes” if the respondent had an affirmative answer and 0 “no” otherwise. Frequency of social media use was categories as “never use”, e.g. which involves respondents do not use any social media, “infrequent user”, e.g. involves respondent who use social media at least once a week, at least once a month or rarely use, “frequent”, e.g. involves respondent who daily use social media, at Wave 1. Duration of social media use was categories as “zero hour”, e.g. involves respondent who do not use in 24 hour, “1-2 hour”, e.g. involves respondent who use social media 1 or 2 hour in last 24 hour, “3 or more hour”, e.g. involves respondent who use social media 3 or more hour in last 24 hour, only for the Wave 2.

### Statistical analysis

To analyze the association between the binary outcome variable and other explanatory variables, the binary logistic regression method was used. The outcome variable ‘depression’ was recoded as 0 “no” for not having or only having minimal symptoms of depression and 1 “yes” for having any form (Mild/Moderate/Severe) of depressive symptoms. Model 1 includes social media use and control for all explanatory variables other than frequency of social media use. Model 2 included the frequency of social media use and controlled for all explanatory variables other than social media for both rounds of survey (Wave 1 and Wave 2 separately).

A longitudinal cross-lagged path analysis was used to examine whether bidirectional relationships exist between social media use and depression. Cross-lagged path analysis is a type of Structural Equation Modelling (SEM) employed to describe reciprocal relationships, or directional influences, between variables and how they influence each other over time. Cross-lagged path models are estimated using longitudinal data where two or more variables are measured on two or more occasions to examine the causal influences between variables [38]. A maximum likelihood estimation procedure was used. The following five models were applied:

- 1: Unidirectional influence of social media use at Wave 1 on social media use at Wave 2.
- 2: Unidirectional influence of social media use at Wave 1 on psychological wellbeing at Wave 2. 3: Unidirectional influence of psychological wellbeing at Wave 1 on psychological wellbeing at Wave 2.
- 4: Unidirectional influence of psychological wellbeing at Wave 1 on social media use at Wave 2 were tested and compared for model fit to determine whether the hypothesized model is the best fitting model. Model fit was examined using Kline's (2010) guidelines according to which good model fit is reached when chi-square value is low and non-significant, comparative fit index (CFI) values are 0.95 or more, root mean square error of approximation (RMSEA) values are 0.05 or less (0.6–0.8 indicates a mediocre model fit), Chi square difference testing and Akaike Information Criterion (AIC) were used to compare the best fit of competing models, whereby the lowest AIC indicated the best fitting model [39]. Statistically significant coefficients within the best fitting model was then examined to interpret specific social media use- psychological wellbeing and psychological wellbeing and social media use. Multiple group structural equation modelling was used to assess whether the cross-lagged associations varied by demographic covariates.

## Results

**Fig. 2:**
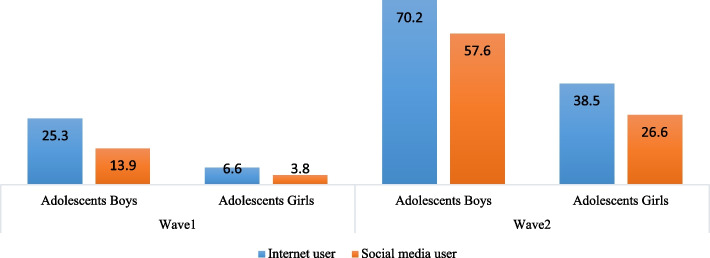
Internet and social media usees (%) among adolecents aged 10-19 ( at wave-1) and aged 13-22 (at wave-2)

**Fig. 3:**
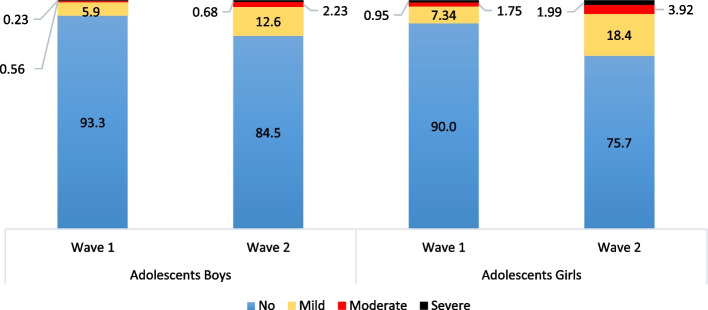
Percentage of adolescents having depressive symtoms at age 10-19 (wave-1) and age 13-22 (wave-2)

Figure 2 depicts the percentage of adolescents' access to the internet and the usage of social media over time. Among adolescent boys, 25.3% and 70.2% access the internet at Wave 1 and Wave 2. The percentage increased from 6.6% to 38.5% among adolescent girls, but there was a big difference in internet access between adolescent boys and girls at both Waves. Social media usage also increased from wave 1 to wave 2. Among adolescent boys, social media use increases from 13.9 % to 57.6% in Wave 1 and Wave 2, respectively, and among adolescent girls, these percentages increase from 3.8% to 26.6%. Figure 3 depicts the depressive symptoms among adolescents. Depressive symptoms in different categories vary from Wave 1 to Wave 2. Among adolescent boys, the percentage of mild depressive symptoms increases from 5.9% to 7.3% from Wave 1 to Wave 2.

The percentage of adolescent boys who suffer from moderate symptoms of depression increases from 0.56% to 1.75%, and 0.23% of boys were having severe depressive symptoms in Wave 1, and it increases to 0.95% in Wave 2. Among adolescent girls also, there was an increment in all forms of depressive symptoms from Wave 1 to Wave 2.

**Fig. 4:**
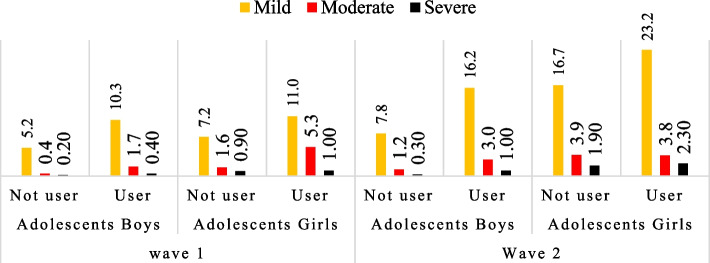
Depressive symptoms (mild to severe) among adolescents aged 10-19 (wave-1) and 13-22(wave-2) by use of social media

In Wave 1, 12.6% of adolescent girls suffer from mild symptoms of depression, and in Wave 2, this percentage increases up to 18.4%, whereas moderate depressive symptoms increase from 2.23% to 3.92% from Wave 1 to Wave 2. The percentage of adolescent girls suffering from severe symptoms of depression increased from 0.68% to 1.99% from Wave 1 to Wave 2. The depression symptoms vary among those who use social media discussed have been discussed in Fig. 4. Adolescent boys, using social media, had mild depressive symptoms, shifted from 10.3% to 16.2%, moderate depressive symptoms shifted from 1.7% to 3.0% from Wave 1 to Wave 2, respectively.

Moreover, severe depressive symptoms increase from 0.41 to 1% at Wave 1 to Wave 2. Among adolescent girls who were using social media, 11.95% suffered from mild symptoms of depression in Wave 1 and became 23.18% at Wave 2. One percentage of adolescent girls suffering from a severe form of depressive symptoms at Wave 1 increases to 2.33% at Wave 2. However, there was a decrement in the moderate form of depressive symptom among adolescent girls using social media from Wave 1 to Wave 2.

The socio-demographic profile of the study population is shown in Table 1. Since this was a follow-up study, it contains a population who participated in both the waves of the survey. About 38.6% of adolescent boys and 18.9% of adolescent girls belong to the age group 10-14 and 61.3% of adolescent boys, and 81% of adolescent girls belong to age group 15-19 at Wave 1. They were followed-up three years later; they shifted to 13-16 and 17-22 years. 23.78% of adolescent boys belong to the more affluent wealth index category, and only 11.37% belong to the poorest category. Furthermore, 24.94% of girls belong to the richer wealth quintile amongst adolescent girls, and 12.32% belong to the poorest wealth quintile. Nearly 82% of adolescent boys were in school at the time of Wave 1 survey, and it decreases to 59.8% at Wave 2, 15.53% adolescents dropped out from school at Wave 1, and 37.86% boys dropped out from school at Wave 2. Among adolescent's girls, 25.52% dropped out of the study at Wave 1. This percentage increases up to 51.72% at Wave 2. 12.64% of adolescent's boys were using substances at Wave 1, and 31.32% uses substances at Wave 2. 24.13% of adolescent boys were worked in paid work at Wave 1, and 55.35% of boys are doing paid work at Wave 2.

**Table1:** Socio-economic and demographic profile of the study population

Background characteristics	Wave1				Wave2			
	Adolescent boy		Adolescent girl		Adolescent boy		Adolescent girl	
Age Group (Wave1, wave2 in parenthesis)								
10-14	38.66	1,712	18.92	1,439	38.66	1,712	18.92	1,439
15-19	61.34	2,716	81.08	6,168	61.34	2,716	81.08	6,168
Religion (Wave1)								
Hindu	84.93	3,729	76.73	5,674	-		-	
Non- Hindu	15.07	699	23.27	1,933	-		-	
Caste (Wave1)								
SC/ST	27.34	1,086	23.97	1,623	-		-	
OBC	54.74	2,505	54.16	4,322	-		-	
Other	17.93	837	21.87	1,662	-		-	
Mother’s Educational level(Wave1)								
Not educated	69.85	2,875	69.69	5,024	-		-	
1-7 years	11.06	503	11.05	832	-		-	
8-9years	8.35	420	8.32	664	-		-	
10 and above years	10.73	630	10.95	1,087	-		-	
Wealth Index (Wave1)								
Poorest	11.37	414	12.32	756	-		-	
Poorer	19.94	699	17.19	1,065	-		-	
Middle	22.26	908	21.1	1,476	-		-	
Richer	23.78	1,195	24.94	2,118	-		-	
Richest	22.66	1,212	24.44	2,192	-		-	
Currently studying								
Current schooling	82.13	3,676	68.43	5,493	59.8	2,725	42.23	3,586
Dropout	15.53	641	25.52	1,666	37.86	1,592	51.72	3,573
Never attend	2.34	111	6.05	448	2.34	111	6.05	448
Substance use								
No	87.36	3,902	99.18	7,549	68.87	3,076	96.31	7,339
Yes	12.64	526	0.82	58	31.13	1,352	3.69	268
Paid work								
No	75.87	951	80.78	1,221	44.65	1,877	26.14	1,870
Yes	24.13	3,477	19.22	6,386	55.35	2,551	73.86	5,737
N		4428		7607		4428		7607

Wave 1(2015-16), Wave 2(2018-19)

The estimate from logistic regression for depression at Wave 1 by different background variables is presented in Table 2. Model 1 explains social media use by controlling other variables, explaining the 5.65% variation in the model. Model 2 includes the frequency of social media use by controlling other variables, explaining the 5.68% variation of the model. Evidence from the analysis shows that adolescents who use social media have a significant relationship with symptoms of depression compared to adolescents who do not use social media (Model 1). The odds of social media users to be depressed were more likely (35.7%) than the non-users.

**Table 2:** Predictors of depressive symptoms (mild to severe) among adolescents aged 10-19 (wave-1): Estimates from logistic regression

**Variables measured at wave-1**	**Model 1**	**Model 2**
	**OR (95% CI)**	**OR (95% CI)**
**Social media use**		
No(Ref.)		
Yes	1.357***(1.099 1.675)	
**Frequency of social media**		
Not user(Ref.)		
Infrequent user		1.226 (0.959 1.566)
Frequently user		1.715***(1.229 2.393)
**Age**		
10-14(Ref.)		
15-19	2.762***(2.425 3.147)	2.76***(2.422 3.144)
**Sex**		
Adolescent Male(Ref.)		
Adolescent Female	1.923***(1.651 2.241)	1.922***(1.65 2.239)
**Mother’s education**		
Not educated (Ref.)		
Educated	1.048 (0.913 1.202)	1.046 (0.912 1.201)
**Wealth index**		
Poorest(Ref.)		
Poorer	0.746**(0.587 0.947)	0.747**(0.588 0.949)
Middle	1.057 (0.848 1.317)	1.058 (0.849 1.318)
Richer	1.029 (0.831 1.274)	1.032 (0.833 1.277)
Richest	1.15 (0.922 1.435)	1.147 (0.919 1.432)
**Current schooling**		
Never(Ref.)		
Current going	0.597***(0.462 0.772)	0.597***(0.462 0.772)
Dropout	0.704**(0.537 0.923)	0.705**(0.538 0.924)
**Substance use**		
No(Ref.)		
Yes	1.575***(1.186 2.091)	1.57***(1.182 2.084)
**Paid work**		
No(Ref.)		
Yes	1.184**(1.007 1.393)	1.187**(1.01 1.396)
Pseudo R2	0.0565	0.0568

***p<0.01; **p<0.05; *p<0.10. *Ref.* Reference category, *CI* Confidence Interval, *OR* Odds Ratio; wave 1 (2015-16)

Adolescents aged 15-19 were two times more likely (OR: 2.762, CI: 2.425-3.147) to have depressive symptoms compared to younger adolescents. Adolescent girls were more likely to have depressive symptoms than adolescent boys. Adolescents who belonged to the poorer wealth quintile were less likely to have depression than their reference category. When discussing current schooling conditions, adolescents currently going to school or dropped out from school were less likely (OR:0.597, CI: 0.462-0.772 and OR:0.704, CI:0.537-0.923) to suffer from depression as compared to those who never attended school. Substance users and those doing any paid work in the last 12 months were 57% and 18% more likely to have depressive symptoms than their counterparts.

The likelihood of depression for a frequent user of social media was represented in Model 2. For adolescents who were frequent social media users, 71% were more likely (OR: 1.715, CI: 1.229-2.393) to have depressive symptoms compared to non-users. Adolescents aged 15-19 years (OR: 2.76; CI: 2.42-3.14) were 2.76 times more likely to have depression than younger adolescents (10-14 years). Female adolescents were more likely (OR: 1.92; CI: 1.65-2.23) to have depression compared to male adolescents. Adolescents who belong to the poorer wealth quintile, less likely (OR: 25.3; CI: 0.58- 0.95) to have depression as compared to poorest wealth quintile adolescents. The odds of depression were 41.3% and 29.5% less likely among adolescents who were currently attending school (OR: 0.597, CI: 0.46-0.77) and who dropped out (OR: 0.705 CI: 0.54-0.92) from school, respectively as compared to adolescents who were never attending school. However, adolescents who use any substance (OR: 1.57; CI: 1.18-2.08) and engaged in paid work (OR: 1.187; CI: 1.01-1.39) were more likely to have depression than their counterparts.

The estimate from logistic regression for depression at Wave 2 by different background variables presented in Table 3. The first model explains the estimate of transition in social media use only, which explains the 3.5% variation in the model. In the second model, duration of use of social media taken into consideration which explains 2.9% variation in the model. The odds of depression were more likely among adolescents who use social media at both the waves of the survey (OR: 1.963; CI: 1.629-2.365), social media users only at Wave 1 (OR: 2.042; CI: 1.284-3.249) and social media users only at Wave 2 as well in comparison to (OR: 1.675; CI: 1.514-1.852) the non-users (Model 1). Adolescents aged 15-19, 32% more likely (OR: 1.325, CI: 1.208-1.454) to have depressive symptoms as compared to younger adolescents. Female adolescents were two times more likely to suffer from depression as compared to male adolescents. Adolescents who were dropped out from school (OR: 1.207, CI: 0.977-1.49), using the substance (OR: 1.445, CI: 1.254-1.666) and done any paid work in the last 12 months (OR: 1.184, CI: 1.075-1.304), more likely to have depressive symptoms as compared to their counterparts.

**Table3:** Predictors of depressive symptoms (mild to severe) among adolescents/young adults aged 13-22 (wave-2): Estimates from logistic regression

**Variables**	**Model1**	**Model2**
	**OR (95% CI)**	**OR (95% CI)**
**Social media use**		
None(Ref.)		
User at both waves	1.963***(1.629 2.365)	
Only at wave 1	2.042***(1.284 3.249)	
Only at wave 2	1.675***(1.514 1.852)	
**Duration of social media use (Wave2)**		
Zero hour(Ref.)		
1-2 hour		1.162**(1.034 1.305)
3 or more hour		1.614***(1.338 1.946)
**Age (Wave1)**		
13-16(Ref.)		
17-22	1.325***(1.208 1.454)	1.411***(1.288 1.546)
**Sex (Wave1)**		
Adolescents Male(Ref.)		
Adolescents Female	2.55***(2.277 2.856)	2.275***(2.036 2.542)
**Mother’s education (Wave1)**		
Not educated (Ref.)		
Educated	0.933 (0.846 1.03)	0.989 (0.897 1.09)
**Wealth Index (Wave1)**		
Poorest(Ref.)		
Poorer	0.949 (0.814 1.105)	0.958 (0.823 1.116)
Middle	0.953 (0.821 1.107)	0.982 (0.846 1.14)
Richer	0.942 (0.814 1.09)	1 (0.865 1.156)
Richest	0.855**(0.731 0.999)	0.973 (0.835 1.135)
**Current schooling (Wave2)**		
Never(Ref.)		
Current going	0.896 (0.722 1.111)	0.98 (0.791 1.214)
Dropout	1.207*(0.977 1.49)	1.257**(1.018 1.551)
**Substance use (Wave2)**		
No(Ref.)		
Yes	1.445***(1.254 1.666)	1.517***(1.317 1.747)
**Paid work (Wave2)**		
No(Ref.)		
Yes	1.184***(1.075 1.304)	1.185***(1.076 1.304)
Pseudo R2	0.035	0.029

***p<0.01; **p<0.05; *p<0.10; *Ref.* Reference category, *CI* Confidence Interval, *OR* Odds Ratio; Wave 1(2015-16); Wave 2(2018-19).

Adolescents using social media for one or two hours as well as three or more hour daily, 16% and 61% more likely (OR: 1.162; CI: 1.034-1.305 and or: 1.614, CI: 1.338-1.946) to have depressive symptoms as compared to their counterpart (Model 2). Older adolescents were 41% more likely (OR: 1.411; CI: 1.288- 1.546) to be depressed compared to younger adolescents. However, female adolescents were two times more likely (OR: 2.275; CI: 2.036-2.542) to have symptoms of depression as compared to male adolescents. Adolescents who were dropped out from the school (OR: 1.257; CI: 1.018-1.551), use substances (OR: 1.517; CI: 1.317-1.747) and also have paid work in the last 12 month (OR: 1.185; CI: 1.076-1.304) were more likely to have depression as compared to their counterparts.

### Measurement variables for depression in Wave 1 & 2

All the measurement variables in the endogenous variables of depression contributed considerably to the model and were statistically significant at *p* < 0.001. The parameter estimates of the indicators of the 'depression*' latent were the highest for A4 (1.24) and A6 (1.24), followed by A3 (1.21), A2 (1.15) and A5 (1.11). Similarly, in Wave 2, the parameter estimates of the indicators of the 'depression**' latent were the highest for B6 (1.36), followed by B4 (1.17) and B3 (1.15) (Table 4).

**Table 4:** Multivariate parameter estimates (β), standard error (SE) and 95% confidence interval (CI) of the measurement variables in the structural equation model

**Codes**	**Indicators**	**Β(SE)**	**95%CI**
Depression*	**Depressive symptoms at wave1**		
A1	Had trouble falling asleep or sleeping too much	**1.00**	
A2	Feeling tired or having little energy	1.15(0.02)***	(1.109 1.191)
A3	Poor appetite or eating too much	1.21(0.022)***	(1.164 1.254)
A4	Trouble concentrating on things	1.24(0.021)***	(1.202 1.285)
A5	Had little interest or pleasure in doing things	1.11(0.019)***	(1.073 1.147)
A6	Feeling down, depressed or hopeless	1.24(0.021)***	(1.196 1.279)
A7	feeling down, depressed or hopeless	0.81(0.015)***	(0.778 0.839)
A8	Been moving or speaking slowly	0.70(0.013)***	(0.672 0.724)
A9	Had thoughts that respondent would be better off dead	0.31(0.009)***	(0.297 0.332)
Depression**	**Depressive symptoms at wave2**		
B1	Had trouble falling asleep or sleeping too much	**1.00**	
B2	Feeling tired or having little energy	1.13(0.024)***	(1.085 1.182)
B3	Poor appetite or eating too much	1.15(0.026)***	(1.099 1.203)
B4	Trouble concentrating on things	1.17(0.024)***	(1.117 1.213)
B5	Had little interest or pleasure in doing things	1.13(0.023)***	(1.087 1.179)
B6	Feeling down, depressed or hopeless	1.36(0.027)***	(1.301 1.409)
B7	feeling down, depressed or hopeless	0.97(0.021)***	(0.924 1.007)
B8	Been moving or speaking slowly	0.79(0.017)***	(0.761 0.829)
B9	Had thoughts that respondent would be better off dead	0.38(0.011)***	)0.359 0.403)
**Model fit statistics**			
Chi-Square	0.000		
**RMSEA**	**0.045**		
CFI	0.889		
TLI	0.870		
SRMR	0.028		
CD	0.426		

^***^ *P* < 0.001; *CD* Coefficient of determination, *CFI* Comparative fit index, *RMSEA* Root mean square error of approximation, *SRMR* Standardized root mean square residual, *TLI* Tucker–Lewis index.

Association between social media usage and depression across two points was analyzed utilizing structural equation modelling (SEM), with maximum likelihood estimation. Cross lagged model (Fig. 4) showed a good fit of data (according to the criteria of RMSEA < 0.06, CFI > 0.90, and TLI > 0.90).

**Fig. 5:**
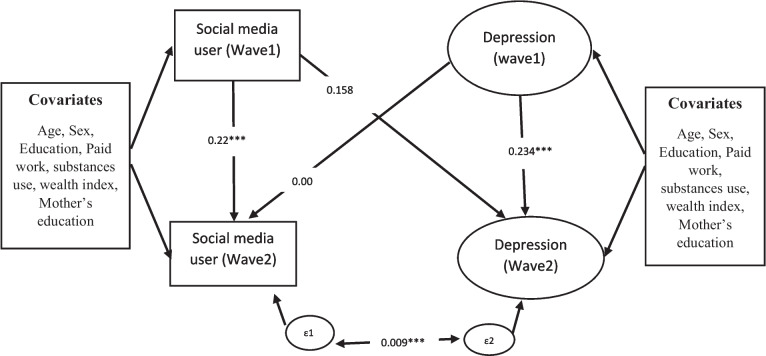
Autoregressive cross-lagged model. Note: Ovals show latent variables; Boxes show observed variables: Error terms are excluded for simplicity. Wave 1 (2015-16), Wave 2(2018-19)

The results show that the path relationship from social media users in Wave 1 [β=0.22, *p*<0.001] were positively associated with social media users in Wave 2. This means a significant increase in social media users among survey participants from Wave 1 to Wave 2. Moreover, depression (Wave 1) [β=0.234, *p*<0.001] was positively related to depression in Wave 2 (Fig. 5).

In other words, depression of Wave 1 has a significant positive effect on depression in Wave 2. However, social media use at Wave 1 was not related to depressive symptoms at Wave 2. Our research question examined whether depressive symptoms among adolescents were related to the usage of social media. Results show that depressive symptoms at Wave 1 were not significantly predicted social media use at Wave 2 and also usage of social media at wave 1 were not significantly predicted depressive symptoms at wave 2 (Table 5).

**Table 5:**
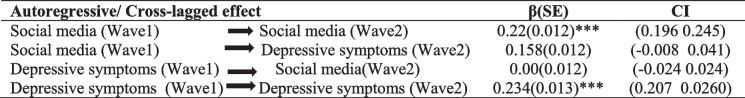
Autoregressive and cross-lagged effects of social media use and depression over the follow-up period

^***^ *P* < 0.001

## Discussion

This study examined the association and relationships between transition in social media use and psychological wellbeing from adolescence to early adulthood among adolescents from Bihar and Uttar Pradesh through cohort data from 2015-16 to 2018-19. The present study showed that the use of the internet increased significantly among adolescent boys and girls from Wave 1 to Wave 2. However, the use of social media use increased more among adolescent boys compared to their girls counterparts. The study also revealed that depression increased considerably among boys and girls from Wave 1 to wave 2.

Many studies have looked upon that social media use is an essential indicator of the risk of mental health problems among adolescents [40]. Social media can significantly impact adolescents' personality, their interpersonal relationships, including events like cyberbullying, normalization of self-harm activities, and suicidal tendencies among them [16]. A similar positive association between social media use and depression was noticed in the present study too. This positive association was held significant in the case of both adolescent girls and boys. Social media use and its harmful effects on adolescents' mental health is a debatable issue. There are studies which reveal that increased time spent on social media was not associated with increased mental health issues across development when examined at the individual level [11, 41] whereas some of the studies have proposed that mental health problems are both cause and result of social media use problems [42]. Studies also advocate that the use of social media in a socially-oriented manner had low significant associations with depressive symptoms. Hence, the frequency of social media use revealing the whole truth is unlikely [31]. However, in the present study, we found a positive relationship between social media and depression, wherein adolescents using social media were more depressed than non-users. This concern stems from several factors, including reduced in-person social interactions [43], addictive tendencies [44] , online bullying [45], heightened social pressure through constant comparisons [46], and the increased exposure to stories about suicide, which can have a harmful influence [47].

Socioeconomic factors played an important role in affecting depression among adolescents in both the waves of the survey. The wealth status and depression were found to have a positive relationship wherein the wealthier adolescents were more likely to be depressed than their respective counterparts. Education acted as a barrier to depression as the adolescents who were educated were found to be less depressed than the uneducated ones. On the other hand, factors like substance use and employment were found to be the facilitators of depression. Age emerged as an important predictor as the adolescents in the age group 10-14 were more vulnerable to depression than the 15-24 years old. Studies have shown that younger adolescents and adults use social media in a more intended manner to transmit social support and communication than older adolescents. It helps explain the changing role of social media in younger adolescents' mental health compared to older adolescents [48]. Gender wise, females were more likely to be depressed compared to their male counterparts. This vulnerability of adolescent girls can be attributed to the existing social structure and the social pressure that they went through in an online world. This finding falls in line with the previous study, which revealed that the association between social media use and depressive symptoms was larger for girls than for boys [14]. In middle-income countries, the relationship between poor psychological wellbeing and screen time or social media use among girls is well documented in the literature [49].

## Conclusion

Though adolescence is a period of several vital shifts in both boys and girls, it is important to identify a few aspects that affect their perspectives and personalities altogether. Social media use is one such aspect that plays an important role in shaping up the ideas and personalities of adolescents, especially the adolescents of the contemporary period who are ruled by an online world. Social media is an integral part of an adolescent's day to day life. Little is known about social media use patterns and how it influences their life in the Indian context. This present study provides insights dynamics of social media use and its longitudinal impact on depressive symptoms among adolescent boys and girls in India. A significant cross-section degree of association is found between social media use and depression among adolescent boys and girls in the study. The present study concludes that factors like age, gender and education showed significant relationships with social media use and depression.

### Limitation

There are a few limitations of this study too. First, the information of variables like type, purpose and time of media use were not available for both the waves of the survey and hence longitudinal inferences could be established. Second, the study is based on a sample of two states, and hence the results may not be generalized for the entire country.

### Recommendation

We suggest and encourage future studies based on time, purpose, and type of social media use and its association with mental health problems other than depression.

### Supplementary Information

**Additional file 1: Table S1. **Multivariate regression coefficients (β), standard error (SE) and 95% confidence interval (CI) of the estimated structural equation model. 

## Data Availability

Data can be accessed through the following link, https://dataverse.harvard.edu/dataset.xhtml?persistentId=doi:10.7910/DVN/ZJPKW5

## Abbreviations

AIC: Akaike Information Criterion
CFI: Comparative Fit Index
PHQ: Patient Health Questionnaire
RMSEA: Root Mean Square Error of Approximation
SEM: Structural Equation Modelling
TLI: Tucker–Lewis Index
UDAYA: Understanding the Lives of Adolescents and Young Adults
