# Supplementary material for: Bidirectional and cross-lag relationship between social media use and psychological wellbeing: evidence from an Indian adolescent cohort study
Source: BMC Public Health. 2024 Jan 26;24:303. doi: 10.1186/s12889-023-17276-1 (PMC10811814; doi:10.1186/s12889-023-17276-1)
Supplement: Supplementary file 1 — Additional file 1: Table S1. Multivariate regression coefficients (β), standard error (SE) and 95% confidence interval (CI) of the estimated structural equation model. [file 12889_2023_17276_MOESM1_ESM.docx]

**Table S1. Multivariate regression coefficients (β), standard error (SE) and 95% confidence interval (CI) of the estimated structural equation model**

| **Variables** | **β(SE)** | **95%CI** |
| --- | --- | --- |
| **Social media (wave1)** |  |  |
| Age(wave1) | 0.04(0.001)*** | (0.033 0.037) |
| Sex (wave1) | -0.19(0.006)*** | (-0.205 -0.181) |
| Wealth Index | 0.01(0.000)*** | (0.008 0.009) |
| Substance user (wave1) | 0.03(0.013)* | (0.003 0.057) |
| Paid work (wave1) | 0.00(0.003) | (-0.012 0.019) |
| Mother’s education | 0.09(0.006)*** | (0.074 0.099) |
| Education of respondent: Dropout | -0.02*(0.013) | (-0.048 0.008) |
| Education of respondent :Continue | 0.06(0.013)*** | (0.029 0.083) |
| **Social media (wave2)** |  |  |
| Social media use(Wave1) | 0.22(0.012)*** | (0.196 0.246) |
| Depression(wave1) | 0.00(0.012) | (-0.024 0.024) |
| Age(wave1) | 0.03(0.001)*** | (0.026 0.033) |
| Sex (wave1) |  | (-0.294 -0.257) |
| Wealth Index | 0.01(0.000)*** | (0.011 0.013) |
| Substance use (wave1) | 0.08(0.012)*** | (0.052 0.102) |
| Paid work(wave1) | 0.04(0.008)*** | (0.023 0.057) |
| Mother’s education | 0.10(0.008)*** | (0.082 0.117) |
| Education : *Dropout | 0.07(0.0198)*** | (0.031 0.109) |
| Education : Continuing | 0.23(0.018)*** | (0.197 0.27) |
| **Depression (wave1)** |  |  |
| Age(wave1) | 0.02(0.001)*** | (0.015 0.021) |
| Sex (wave1) | 0.09(0.007)*** | (0.076 0.104) |
| Wealth Index | 0(0.000)** | (0 0.002) |
| Substance use (wave1) | 0.03(0.016) | (-0.001 0.063) |
| Paid work(wave1) | 0.04(0.009)*** | (0.022 0.058) |
| Mother’s education | 0(0.007) | (-0.018 0.012) |
| Education : *Dropout | 0.02(0.016) | (-0.018 0.048) |
| Education : Continuing | 0.02(0.016) | (-0.013 0.05) |
| **Depression (wave2)** |  |  |
| Social media use (wave1) | 0.158(0.012) | (-0.008 0.041) |
| Depression (wave1) | 0.234(0.013)*** | (0.207 0.0260) |
| Age(wave1) | 0.011(0.001)*** | (0.007 0.014) |
| Sex (wave1) | 0.133(0.009)*** | (0.114 0.152) |
| Wealth Index | -0.001(0.000)* | (-0.002 -0.000) |
| Substance use (wave1) | 0.05(0.012)*** | (0.024 0.073) |
| Paid work(wave1) | 0.023(.008)** | (0.006 0.046) |
| Mother’s education | -0.003(0.008) | (-0.021 0.013) |
| Education : *Dropout | -0.00(0.019) | (-0.034 0.042) |
| Education : Continuing | -0.005(0.018) | (-0.042 0.030) |
| **Cov (social media user (wave2), depression (wave2))** | **0.009(0.001)***** | **(0.006 0.013)** |

Note: Wave 1(2015-16), Wave 2(2018-19)
